# Bedside brochoscopy a concern

**DOI:** 10.1186/1753-6561-5-S6-P73

**Published:** 2011-06-29

**Authors:** R Rao

**Affiliations:** 1 Microbiology, Apollo Hospital,Hyderabad,Ap.India, Hyderabad, India

## Introduction / objectives

Bedside bronchoscopy has become a common procedure for the increasing number of patients getting admitted to intensive care. Process flow for the bronchoscope is a concern when it’s done away from its workstation. We describe here an easy to follow methodology for this bedside procedure.

## Methods

All requests for the procedure were routed to the bronchoscope unit. The bronchoscopes (consultant’s personal device) had to be submitted to the unit two hours before the procedure. A written protocol for its processing, disnfecting and transported (in sterile covers ) to the bedside was documented. Training was imparted to all the related personnel. Check list was created for the same. Compliance study for the process flow was carried out regularly. All bedside bronhoscopy specimens were cultured, monitored, clinically correlated and analyzed.

## Results

Rate of isolation pseudomonas and acinetbacter came done drastically. Pre wash of the scopes before the procedure were negative for acid fast bacilli in all the samples. Compliance to adherence to protocol was 99%.

## Conclusion

Iatrogenic infections with resistant organisms can be reduced markedly if simple clear protocols are documented, montered and analyzed .Regular follow up is required to sustain the compliance.

## Disclosure of interest

None declared.

